# Trace Element Supplementation of Livestock in New Zealand: Meeting the Challenges of Free-Range Grazing Systems

**DOI:** 10.1155/2012/639472

**Published:** 2012-12-20

**Authors:** Neville D. Grace, Scott O. Knowles

**Affiliations:** ^1^26 Williams Road, RD 4, Palmerston North 4474, New Zealand; ^2^Food and Bio-Based Products Group, Grasslands Research Centre, AgResearch Limited, Private Bag 11008, Palmerston North 4442, New Zealand

## Abstract

Managing the mineral nutrition of free-range grazing livestock can be challenging. On farms where grazing animals are infrequently yarded, there are limited opportunities to administer trace element supplements via feeds and concentrates. In New Zealand, where the majority of sheep, cattle, and deer graze pasture year round, inadequate intake of cobalt, copper, iodine and selenium is prevalent. Scientists and farmers have developed efficient strategies to monitor and treat these dietary deficiencies. Supplementation methods suited to grazing livestock include long-acting injections, slow-release intraruminal boluses, trace element-amended fertilisers, and reticulated water supplies on dairy farms.

## 1. Introduction

Grazing land for livestock has expanded sixfold since 1800 and now covers more than 25% of the earth's ice-free land surface (about 3400 million ha [1]). This includes large areas where previously there has been little grazing, such as North and South America and Australia. Extensive (on marginal drylands) and intensive (on improved pasture) grazing supports 400 million cattle and 600 million sheep and goats [2]. The latter system delivers about one-fifth of the world's supply of beef, sheep, and goat meat, which is many times greater production than from industrialised farming systems characterised by feedlots. Managing the mineral nutrition of grazing livestock can be challenging. On farms where animals are infrequently yarded, there are limited opportunities to administer trace element supplements via feeds and concentrates.

Livestock farming in New Zealand is pastoral, based on ryegrass and clover swards that thrive in a temperate environment with substantial rainfall. Grazing management is efficient and low cost [3], in part because animals remain outdoors throughout the year. The industry currently comprises about 6 million dairy cows, 4 million beef cattle, 32 million sheep, and 1 million deer grazed on 9.3 million hectares of grassland across flat, rolling, and steep hill country [4]. Some feed supplements such as grass and maize silages and palm kernel expeller (PKE) are provided for the dairy herds but contribute less than 15% of their annualised energy intake. Routine management of sheep and beef cattle requires only infrequent mustering and yarding, in order to carry out husbandry tasks such as the establishment of mating groups, pregnancy diagnosis, tailing or marking, shearing, and weaning.

While pasture and forages generally provide sufficient mineral intakes for livestock, deficiencies can occur. The elemental composition of pasture is affected by underlying soil composition, which is itself a consequence of the origin and geochemistry of the local landmass. In New Zealand, soil composition varies markedly with location, and national surveys determined long ago that some regions could not support good animal production. For example, selenium (Se) and cobalt (Co) deficiencies were observed in sheep and cattle grazing pastures sown on volcanic soils [5, 6]. Since then, numerous animal response studies, typically measuring growth rate or the presence and absence of clinical signs and symptoms, have shown that livestock in New Zealand are at significant risk of inadequate dietary intake of four trace elements: Se, Co, copper (Cu), and iodine (I) [7]. Deficiencies of other essential elements observed elsewhere (e.g., zinc and manganese [8, 9]) have not been documented in New Zealand. 

The mineral status of livestock is usually determined by means of biochemical criteria and reference ranges established for the particular species and farm management system. Commercial animal health laboratories and veterinary clinics in New Zealand recognise the following minimum values as indicative of adequate trace element status: blood Se 250 nmol/L; serum vitamin B_12_ 350 (sheep) 120 (cattle) pmol/L; serum Cu 8 *μ*mol/L; liver Cu 100 *μ*mol/kg fresh tissue [7]. A reliable biochemical criterion for I status has not yet been determined [10]. 

Rather than attempting year round supplementation of deficient elements, the timing and delivery to grazing animals should focus on vulnerable phases of the lifecycle, particularly reproductive performance of dams and the growth of young animals. For example, premating treatment of ewes with Se and I supplements can improve fecundity in terms of increased lambing percentage and decreased perinatal mortality [11]. For maximum benefit to offspring, sometimes the best approach is to treat the dam and take advantage of the fact that Se, Co, Cu, and I readily cross the placenta [12], and Se, Co, and I are also secreted into milk. This can increase the trace element status of the developing foetus, newborn, and suckling young for variable periods up to weaning [13, 14].

When supplementation is warranted, it should suit the species, farm policy, facilities, and budget. A key consideration is the duration of efficacy of available supplement products. This varies from 1 to 12 months or more, depending on chemical form of the trace element, its physical presentation, and its route of administration. Broadly, the measures can be categorised as being short-acting with fast absorption and quick utilisation (see Section 2), or long-acting with slower absorption that provides extended supplementation (see Section 3). 

**Figure 1:**
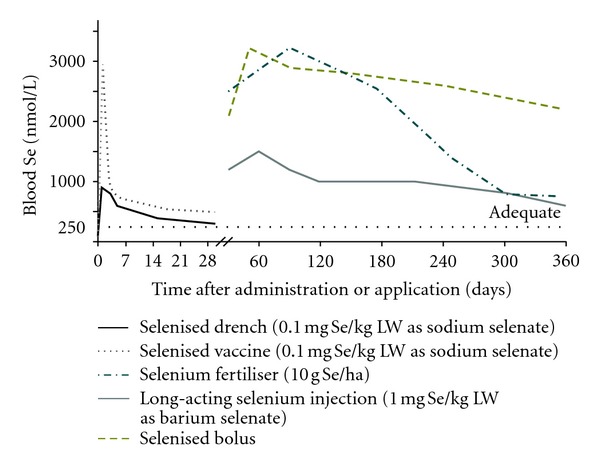
Comparison of the effects of administration or application of a selenised oral drench, a selenised vaccine, a Se-amended fertiliser, a Se-containing intraruminal bolus, and a long-acting Se injection on the blood Se concentrations of sheep. Blood Se >250 nmol/L indicates adequate Se status, above which animals are unlikely to respond to supplementation in terms of improved performance or production.

**Figure 2:**
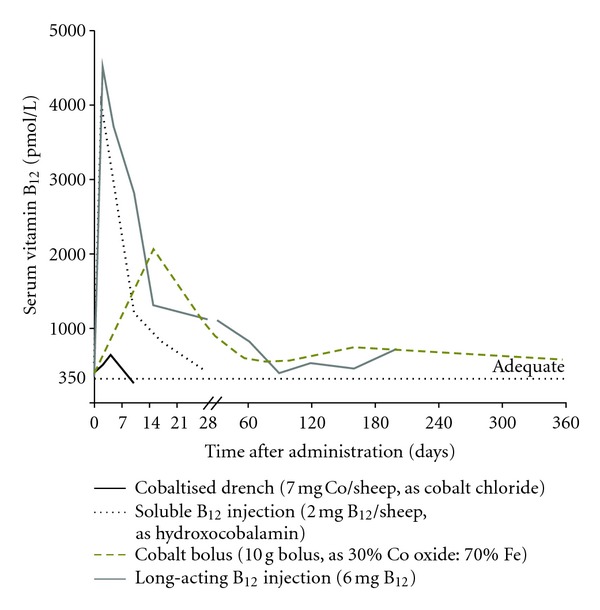
Comparison of the effects of administration of a cobaltised oral drench, a soluble vitamin B_12_ injection, a Co-containing intraruminal bolus, and a long-acting vitamin B_12_ injection on the serum vitamin B_12_ concentrations of sheep. Serum vitamin B_12_ >350 pmol/L indicates adequate vitamin B_12_ status, above which animals are unlikely to respond to supplementation in terms of improved performance or production.

The efficacies of options for supplementing livestock with Se and Co are compared in Figures 1 and 2, [15, 16]. Sustained results are obtained with trace element-amended fertiliser, intraruminal boluses, and injections formulated to create a subcutaneous or intramuscular depot that provides slow and controlled release. In this paper, we emphasise those methods and review their use in some detail. 

## 2. Supplements Providing Short-Term Efficacy

Widely available trace element supplements having fast-acting, short-term efficacy are best suited to farming operations where animals are closely managed or handled frequently. They can be impractical for free-range grazing livestock because doses must be repeatedly administered throughout the period of risk of dietary deficiency. This can be a demanding exercise with concomitant high labour costs. Forms of the supplements are usually chemically simple, water soluble, and readily absorbed. Typical methods for delivering supplemental elements, primarily via oral administration, are described briefly below.

### 2.1. Oral Drenches and Short-Acting Injections

Many oral liquid dose products (“drenches”) formulated as anthelmintics contain Co, Se, or Cu [17]. Efficacy of the minerals is typically 1 week for Co, 5-6 weeks for Se, and several weeks for Cu. Some injectable clostridial vaccines contain water soluble Se or vitamin B_12_. As efficacy is only 4–7 weeks (or longer under some conditions [18]), several injections are required to adequately supplement a lamb for the 6–8 month period of birth through weaning to slaughter weight [19]. When trace elements are included in drenches and vaccines, their timing and frequency of administration is dictated by protocols for disease and endoparasite control. This is unlikely to be in phase with the animal's physiological and metabolic requirements for the elements.

Exceptions can be considered for pregnant ewes fed brassica crops such as kale, swedes, and rutabaga that are low in I concentration and contain I-sequestering goitrogens that reduce circulating levels of iodinated thyroxine hormones. Drenching twice, at 8 and 4 weeks prior to lambing with 200 mg I can be an effective, albeit laborious, approach to preventing I deficiency in newborn lambs [20]. 

### 2.2. Solidified Free-Choice Products

Compressed salt blocks and molasses licks containing trace elements are simple methods of providing supplementation that do not necessarily require yarding of animals. However, they are not completely satisfactory because many behavioural factors influence interest and craving, and therefore not all animals will receive adequate intake [21].

### 2.3. Water Supply

Dispensing soluble minerals into a metered water supply suits farms where animals have daily access to water troughs and drinking can be assured. Consumption will vary depending on air temperature and moisture content of the pasture. Alert farmers are able to adjust the mechanical dispenser to accommodate changes in environmental conditions and manage their herd's trace element intake. Many varieties of mineral premixes are available, and some are differentiated by chemical forms that resist precipitation and volatilisation. This method is in use on half of New Zealand's 10,000 dairy farms yet, remarkably, there is very little peer-reviewed research describing delivery of the elements to trough drinking water or the efficacy of ingested elements to affect the biochemical criteria, performance and production of animals. 

### 2.4. Feed Supplements

Adding trace elements to feed supplements is standard practice where rations are fed indoors or on feedlots. While there is no such control over diet and intake on most grazing operations that raise sheep and beef cattle, dairying is more flexible. Trace and macro elements can be added to conserved hay and silages, PKE, and grain-based concentrates when cows are fed on feedpads. Relative to pasture, feeding supplements to cows is expensive, so the practice tends to be limited to about 15% of the annualised energy intake.

## 3. Supplements Providing Long-Term Efficacy

Trace element supplements having long-term efficacy reduce the labour cost of repeatedly mustering animals and administering treatments. These products tend to be chemically sophisticated and more expensive than short-acting versions. Some are formulated to create a subcutaneous or intramuscular depot that dissipates slowly and provides extended release (see Section 3.1). Others are heavy self-contained boluses for oral administration that rest in the reticulorumen and dissolve over time (see Section 3.2). Indirect supplementation of animals is also possible for some elements by way of trace element-amended fertilisers. On the hilly terrain of New Zealand, these are often applied by helicopter or aerobatic “crop duster” airplanes (see Section 3.3). 

### 3.1. Depot-Forming Injections

Products that create a persistent depot are usually injected into the animal's anterior neck region, so that there is little risk of carry-over to the dressed carcass. Technologies to deliver controlled release of trace elements or other micronutrients include lipidic or water-insoluble chemical forms as well as encapsulation of active compounds in liposomes or solidified resins. Preferred products allow dose or depot size to be varied, in order to match the magnitude of the deficiency or the shorter period of protection required for animals intended for slaughter.

#### 3.1.1. Cobalt (Vitamin B_12_)

**Figure 3:**
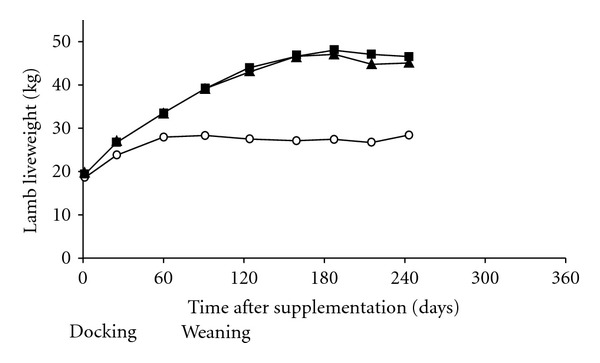
Effect of no treatment (◯) or subcutaneous injection of 3 mg (▲) or 6 mg (■) microencapsulated vitamin B_12_ (0.15 and 0.30 mg/kg LW) administered to Co-deficient lambs at docking time on liveweight [22].

**Figure 4:**
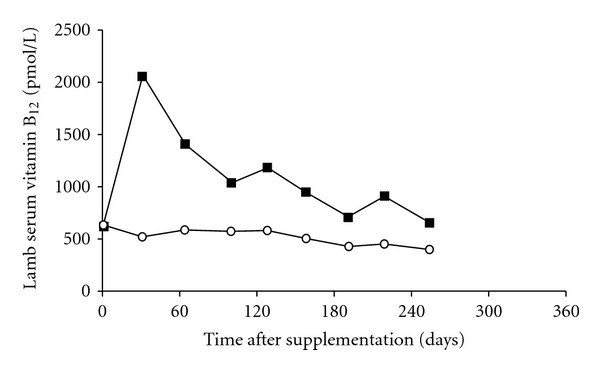
Effect of no treatment (◯) or subcutaneous injection of 3 mg microencapsulated vitamin B_12_ (about 0.20 mg/kg LW) (■) administered to Co-adequate lambs at docking time on serum vitamin B_12_ concentrations [23].

Cobalt is a constituent of vitamin B_12_, thus Co deficiency is really a vitamin B_12_ deficiency. The microbes in a mature rumen convert dietary Co into vitamin B_12_, which the animal absorbs. Very young, preruminant livestock require preformed vitamin B_12_ in milk, milk replacer diets, or supplements. A long-acting injectable form of vitamin B_12_ has been developed that contains B_12_ microencapsulated in lactide-glycolide polymer. It is especially suited to young lambs, which are most sensitive to Co deficiency. A dose rate of about 0.20 mg B_12_/kg LW will increase and maintain the animal's vitamin B_12_ status for 6–8 months. Supplementation can markedly reduce mortality and improve the weight gain of deficient lambs. Dramatic effects on 3–5-week-old lambs supplemented at the time of docking, from a flock grazing Co-deficient pastures, are illustrated in Figures 3 and 4. A threefold increase in liver vitamin B_12_ reserves was also maintained for at least 124 days [22]. Early identification and treatment of Co/vitamin B_12_ deficiency pays dividends, because lambs will achieve slaughter weights of 32–42 kg sooner. Although Co deficiency is prevalent among flocks in New Zealand, it has not yet been documented in young cattle.

#### 3.1.2. Selenium

**Figure 5:**
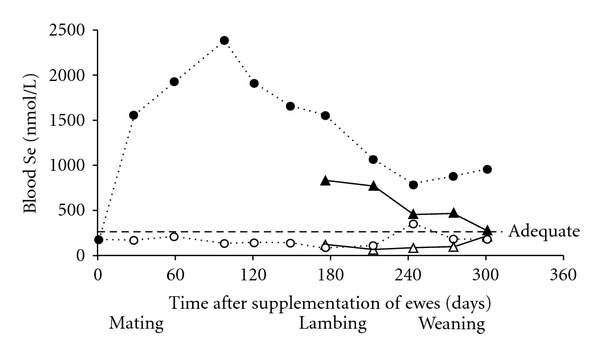
Effect of no treatment (◯) or subcutaneous injection of 60 mg Se as barium selenate (about 1 mg Se/kg LW) (●) administered to ewes 4 weeks prior to mating on blood Se concentrations of ewes (◯, ●) and their lambs (∆, ▲) from birth to weaning. Adequate Se status is indicated by blood Se >250 nmol/L [12].

Selenium deficiency is common among sheep and cattle on the central North Island and east coast of the South Island of New Zealand. This has spurred the use of a range of options for prevention and treatment. Injectable products containing insoluble barium selenate create a depot in the tissue that provides long-term supplementation. A dose rate of 0.5–1.0 mg Se/kg LW suits most ruminant species and will increase and maintain selenium status for 10–18 months [24]. Selenium crosses the placenta, so it is convenient to administer a single injection to ewes 4 weeks prior to mating in order to address fertility issues, and to increase ewe Se status and that of her lamb until weaning or slaughter at 16–20 weeks of age (Figure 5) [12]. Alternatively, lambs at risk of Se deficiency affecting their growth can be supplemented directly at 3–5 weeks of age at the time of docking. Cows are usually treated prior to mating or early during gestation; their response in terms of blood Se concentration is similar to ewes [25]. Young cattle are supplemented after weaning (5-6 months of age) so that a single injection will suffice until slaughter at 18–20 months of age. 

#### 3.1.3. Copper

Injectable Cu products do not have true controlled-release behaviour. Instead, their efficacy is extended by accumulation of excess Cu in liver, which provides reserves for periods of insufficient dietary intake. Typical formulations contain soluble calcium copper edetate injected subcutaneously at a dose rate of 0.3–1.0 mg Cu/kg LW to provide 1-2 months of cover. The Cu is absorbed quickly and overdose can cause poisoning. Sheep are more sensitive to this toxicity than cattle. Injectable Cu supplements can be an effective approach for cattle; however, in most situations cows must be injected several times during gestation to maintain their Cu status and that of neonatal calves [26].

#### 3.1.4. Iodine

Iodine covalently bound to polyunsaturated fatty acids (“iodised oil”) is water-insoluble and upon subcutaneous or intramuscular injection forms a depot that is slow to degrade. A dose rate of 5–7 mg I/kg LW maintains I status for 8 months or more [27]. 

**Figure 6:**
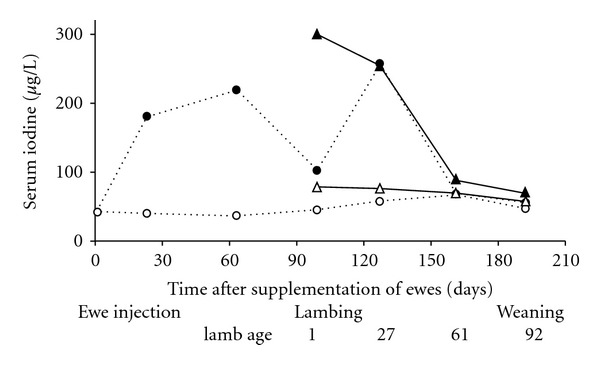
Effect of no treatment (◯) or intramuscular injection of 400 mg iodised oil (about 7 mg I/kg LW) (●) administered to ewes during early gestation on the concentrations of serum total I in ewes (◯, ●) and their lambs (∆, ▲). The percentage perinatal mortality and the thyroid-weight : birthweight ratio (g/kg) in lambs from the unsupplemented and I-supplemented ewes were 27% versus 16% and 0.70 versus 0.27, respectively. Ratios >0.4 g/kg indicate that a flock is at risk of deficiency and is likely to respond to I supplementation [10, 13].

Iodine deficiency in New Zealand is most often associated with feeding brassica crops to pregnant ewes during the winter when pasture growth is insufficient to meet the animals' energy requirement. The low I concentration and goitrogen content of some brassica species can cause thyroid gland enlargement (goitre) in 50% or more of newborn lambs. Farm operations will supplement flocks with iodised oil early during gestation, for instance at 4 weeks prior to mating or when ewes are mustered for ultrasound scanning to determine pregnancy status. This raises serum total I concentrations, prevents goitre (i.e., the thyroid : birthweight ratio is less than 0.4 g/kg) and reduces deficiency-associated perinatal mortality regardless of diets fed (Figure 6) [28, 29]. 

Iodine deficiency has not been documented in cattle in New Zealand. Studies investigating the metabolism of iodine in dairy cows showed that supplementation with iodised oil at 5 mg I/kg LW increased serum total I concentrations for about 100 days [30] as well as increased milk I concentrations 8–10 fold (i.e., 20 versus 200 *μ*g I/L) [31].

### 3.2. Intraruminal Boluses

Intraruminal boluses (sometimes called pellets, bullets or capsules) are heavy self-contained devices designed to be administered orally and remain in the reticulorumen for the life of the animal, or at least until fully dissolved. They can be made from solid metals, resins, or glasses that contain one or more trace elements and are manufactured in sizes to match species and livestock class [32–34]. As efficacy is usually long (up to 12 months), boluses seem ideally suited to free-range grazing operations. In practice, the safe and quick administration of oral boluses is a skilled job requiring good raceway, yarding, and restraint facilities. With the exception of the CuO described below, boluses are no longer widely utilised in New Zealand; long-acting injectable products are preferred. 

#### 3.2.1. Cobalt, Selenium, and Iodine

Cobalt is an ingredient in several types of bolus products. The most concentrated formulation contains 30% Co_3_O_4_ plus 70% iron and has an efficacy of up to 12 months. A 10 g and 30 g bolus is used for lambs and calves, respectively. In some situations, it can be regurgitated and lost or, within the reticulorumen, it can become coated with a deposit of calcium phosphate and fail to dissolve properly [35]. The simplest Se bolus contains 5–10% Se plus 90–95% iron that dissolves slowly over 12 or more months. Ewes may be supplemented with 10 g at 3-4 weeks prior to mating, while cows should receive 2 × 30 g during early gestation [32]. Soluble glass boluses containing I [36] and other elements [37, 38] have been developed in the United Kingdom, but are not available for use in New Zealand.

#### 3.2.2. Copper

An effective and popular Cu bolus is a gelatine capsule containing small CuO wire particles, sometimes called “copper needles” [14, 39]. The dose rate is 0.05–0.10 g/kg LW. The particles stick in the reticulorumen from which they are dislodged slowly to dissolve in the acidic gastric fluid of the abomasum, then the Cu is absorbed in the small intestine. A single 20 g bolus of CuO administered to cows provides the dietary intake equivalent of about 100 mg Cu per day [40]. Efficacy, which includes accumulation and release of Cu reserves in liver, is 6–9 months. This method of supplementation has minimal risk of overdose and poisoning because the bioavailability of Cu as CuO is very low [41]. 

**Figure 7:**
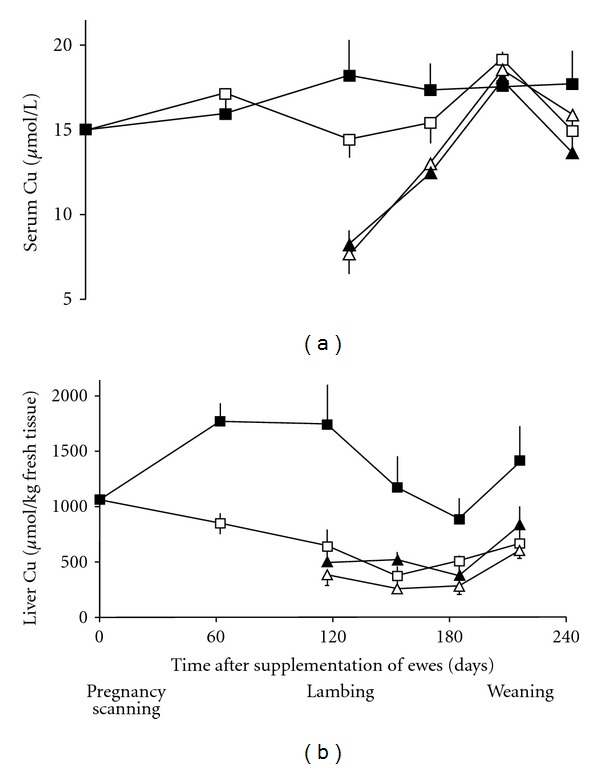
Effect of no treatment (□) or bolus administration of 5 g CuO wire particles (about 0.1 g Cu/kg LW) (■) administered to pregnant ewes early in gestation on the concentrations of Cu in (a) serum, and (b) liver of ewes (□, ■) and their lambs (∆, ▲). Although the flock was not initially Cu deficient, efficacy of CuO particles was demonstrated by changes in liver Cu concentrations [14].

A Cu bolus given to ewes and dairy cows during early gestation will increase Cu reserves of the foetus and Cu status of the newborn. The effect in ewes and their lambs from birth to weaning is shown in Figure 7 [14]. A similar pattern has been observed in cattle [42]. These results illustrate the importance of using the correct tissue and biochemical criterion to assess the efficacy of a mineral supplement [43]. While serum Cu changed little in these adequate-status animals, there was a substantial increase in liver Cu concentration, an effect that is consistently seen elsewhere [44]. 

Compared to parenteral supplementation, nutrients derived from a bolus are subject to some of the same environmental influences as nutrients ingested from the diet. In the case of Cu, excessive dietary molybdenum (Mo) and sulphur can form insoluble Cu thiomolybdates in the rumen. These interfere with the animal's absorption and hepatic storage of Cu. Pastures that are high in Mo content (i.e., >3 mg Mo/kg DM) can reduce the efficacy of oral Cu supplements by 30–50% [45, 46].

### 3.3. Trace Element-Amended Fertilisers

The maintenance of productive pastures requires input of fertilisers, particularly on dairy farms. Regularly scheduled applications provide opportunities to use the fertiliser as a carrier for trace elements, allowing them to be evenly distributed over pastures for the benefit of grazing animals. Uptake by herbage is influenced by application rate, soil composition, and botanical diversity. 

This approach is widely practiced in New Zealand with Se, Cu, and Co. It can markedly increase the dietary intakes of grazing livestock, but requires planning and careful management of animal movements. For 3-4 weeks following application, trace element-amended fertiliser should be allowed to settle into the soil and start to be accumulated in herbage. Animals should not graze the treated pastures during this time. When they graze the enriched pastures during the subsequent 3-4 months, they absorb and retain excess intake. For example, high dietary intake of Se is stored in body proteins as seleno amino acids, while Cu, and to a lesser extent Co/vitamin B_12_, is stored in the liver. These reserves are utilised later when dietary intake is less than metabolic demand. Annual applications of trace element-amended fertiliser can prevent Se and Cu deficiencies for at least 12 months and Co deficiencies in lambs for 6-7 months. 

#### 3.3.1. Cobalt

**Figure 8:**
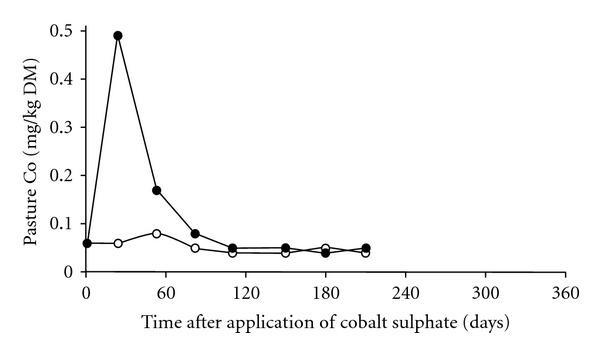
The effect of none (◯) or 350 g cobalt sulphate per hectare (70 g Co/ha) (●), applied as a solid, on the concentrations of Co in pastures. Adequate intake of Co requires herbage Co >0.06 mg/kg DM for cattle and >0.10 mg/kg DM for lambs [48].

Recommended application rate is 70 g Co/ha, usually as cobalt sulphate applied annually in the spring to provide high Co pastures for weaned lambs in the summer. Concentrations of Co in herbage can be increased from 0.06 to 0.50 mg Co/kg DM (Figure 8). Grazing this pasture for 3 months will prevent Co deficiency in lambs for a further 4 months, maintaining serum vitamin B_12_ concentrations >350 pmol/L [47, 48].

#### 3.3.2. Selenium

**Figure 9:**
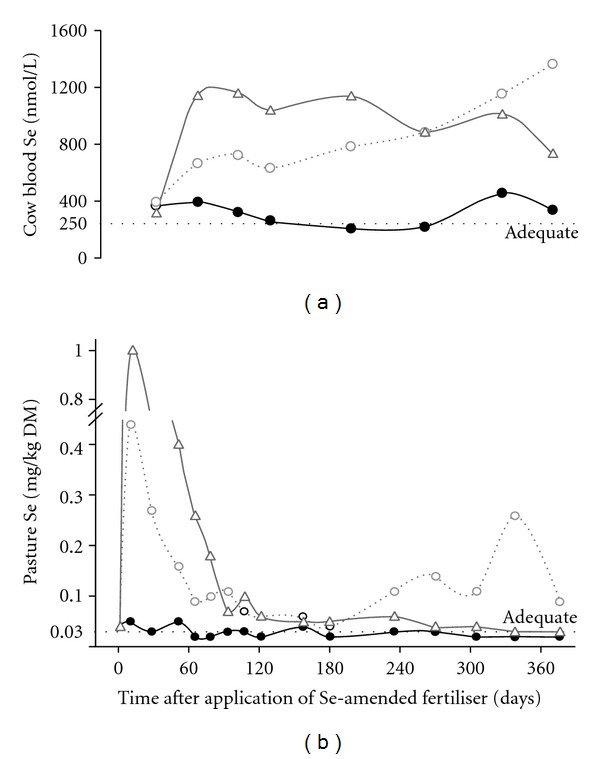
Effect of none (●) or 10 g Se/ha as sodium selenate (∆) or sodium selenate plus barium selenate (◯) applied to pastures grazed by dairy cows on the concentrations of Se in (a) blood, and (b) pasture herbage. Adequate Se status is indicated by blood Se >250 nmol/L and herbage Se >0.03 mg/kg DM [49].

Recommended application rate is 10 g Se/ha, usually carried in semisoluble “prills” applied annually during the autumn or the spring. Herbage Se concentrations can increase 20–35 fold (e.g., from 0.03 to 1.0 mg Se/kg DM) during the first month before falling sharply then more slowly over the next 70–120 days [49, 50]. The Se status of grazing livestock responds to this high Se intake. The changes in blood Se concentrations of dairy cows on treated pastures are shown in Figure 9. A similar response has been observed in sheep [51]. 

#### 3.3.3. Copper and Iodine

Application rate for Cu depends on the livestock being farmed. The range is 1.5–3 kg Cu/ha, usually as copper sulphate applied in the autumn or the spring. The lesser amount will increase pasture Cu concentrations from 4 to 25 mg/kg DM and is suitable for sheep. As the dietary Cu requirement of cattle and deer is twice that of sheep (i.e., 10 versus 5 mg Cu/kg DM), a higher application rate is used for these species. The time-course and concentration profile of Cu in treated pastures is usually similar to that observed for Co and Se [52, 53], although the method is known to be less effective with some herbage species and soil types [46]. 

Iodine-amended fertilisers are not routinely used. It has been observed that the uptake of I by plants is not very efficient and can be highly variable, leading to unpredictable animal responses [54].

## 4. Summary

Most of the 43 million head of ruminant livestock in New Zealand receive a high proportion of their nutrient requirements from grazed pastures and are mustered and yarded infrequently. This poses challenges that are different to industrialised systems or feedlots where animals are handled daily and fed formulated rations. The development and use of long-acting injectable products, slow-release intraruminal boluses and annually applied trace element-amended fertilisers enable sheep and cattle to be supplemented once a year. Timing can target the vulnerable young animals or ewes and cows prior to mating or early in gestation. Alternative products, such as oral drenches and injections that contain water soluble salts with short-acting efficacy, can be cheaper to formulate and buy, but total labour costs are usually greater.

Whether for free-range or feedlot animals, trace element supplementation programmes all carry some hazard of misapplication, overdose, and toxicity. Risks are low for the controlled quantities used for slow-release injections and the fixed dose sizes of intraruminal boluses, but increasing use of feed supplements such as conserved hay and silages, PKE, and grain-based concentrates brings new complications. Inattention to cumulative intakes of minerals from feed and supplement sources may be responsible for numerous cases of chronic Cu toxicity among dairy cows (liver Cu concentration >4000 *μ*mol/kg fresh tissue), due in part to the 25–30 mg Cu/kg DM in PKE [43, 55]. Thus, our understanding and implementation of trace element supplementation must keep pace with farming trends.
